# Perceived Impacts of Climate Change in Pastoralist Regions of Ethiopia: A Qualitative Study Applying the Concept of One Health

**DOI:** 10.3390/ijerph22020257

**Published:** 2025-02-11

**Authors:** Mulugeta Tamire, Siobhan M. Mor, Matthew Baylis, Mirgissa Kaba

**Affiliations:** 1 Department of Preventive Medicine, School of Public Health, Addis Ababa University, Addis Ababa P.O. Box 9086, Ethiopia; mirgissk@yahoo.com; 2 International Livestock Research Institute (ILRI), Addis Ababa P.O. Box 5689, Ethiopia; smor@liverpool.ac.uk; 3 Institute of Infection, Veterinary and Ecological Sciences, University of Liverpool, Leahurst Campus, Neston CH64 7TE, UK; matthew.baylis@liverpool.ac.uk

**Keywords:** climate change, pastoralist perception, one health, food security, Ethiopia

## Abstract

Ethiopia is highly vulnerable to the effects of climate change, with the increasing geographic extent, intensity, and frequency of drought. This study aimed to examine how pastoralist communities understand climate change and its impacts. We conducted a qualitative study among pastoral communities in Ethiopia using focus group discussions with community representatives and key informant interviews with human and animal health and agriculture experts. The collected data were analyzed using qualitative content analysis. Participants viewed deforestation and population growth as the main causes of climate change. They found that climate change caused high temperatures, a shortage of rainfall, and drought. These changes affected the environment, food security, and animal health, impacting their livelihoods, health, and social systems. Coping strategies included engaging in new economic activities, environmental recovery attempts, migration, and seeking food aid for survival. They suggested providing food aid, improving access to human and animal health care, and implementing early warning systems at the community level. The pastoralists perceived that climate change destroyed the natural environment, increased food insecurity, and negatively affected social systems and health. Collaborative actions are needed to mitigate these effects, initiate local environmental adaptation mechanisms, enhance water and food security, and improve animal and human health services.

## 1. Introduction

Anthropogenic climate change presents an existential threat to humankind in the twenty-first century [1]. Effects of climate change are already being felt around the world and include increases in temperature, erratic rainfall and unpredictability of seasonal rain, and increased frequency of extreme weather events such as drought [2,3]. Although no country is immune to its effects, climate change disproportionately affects low-income countries with poor technological advancements needed to monitor and reduce greenhouse gases and limited coping capacities [4]. Typically, an equatorial location also predisposes low-income countries to lower natural temperature variability and thus greater changes in the occurrence of temperature extremes induced by global warming [5,6].

Ethiopia is the second most populous country in Africa, with a population of over 120 million [7]. Like other Sub-Saharan African countries, the country is highly vulnerable to the effects of climate change [6]. It is one of the world’s most drought-prone countries, with an increasing geographic extent, intensity, and frequency of droughts during the last few decades. The 2015 El Niño-induced drought caused food insecurity among 10.2 million people, one of the highest on record at the time [8]. The country has also experienced flooding, leading to the displacement of communities and destruction of infrastructure. Deforestation, soil degradation, and climate change exacerbate these extreme weather events [9]. Rising temperatures and desertification in the lowlands of Ethiopia is also expanding, due to the country’s location in the Sahara desert’s influence area [10].

Pastoral livelihood and food security is founded on livestock, and this remains an integral part of life in such settings. The pathways through which livestock contributes to pastoralist livelihoods and food security are diverse and multifaceted, making these animals an integral part of life [11]. Rearing and trading livestock has been a long-established practice among pastoralist societies, providing them with essential resources, such as milk, meat, skins, hides, and other byproducts. Livestock also serves as a valuable source of income through the sale of animals and their products in local markets, enabling pastoralists to purchase other necessities such as household items and agricultural inputs. They also offer social, cultural, and symbolic values that are deeply intertwined with the lives of pastoralists [11,12].

However, climate change has been disrupting the ecosystems through altered rainfall patterns, increased temperatures, and changes in the timing of seasonal events. This poses a risk to the survival of animals that might create a double burden for pastoralists whose lives are dependent on their livestock [13,14]. Studies have shown that climate change has negatively impacted livestock production among pastoralists in the Somali and Borena regions of Ethiopia, leading to a decline in animal populations due to feed and water shortages, reduced productivity, decreased mature weight or longer time to reach mature weight [14,15]. Another study conducted among Hammer pastoralists also observed the scarcity of water and grazing land caused by climate change. This has impacted livestock production, leading to negative health outcomes such as malnutrition, especially among vulnerable groups such as children [16].

The One Health concept is a recognized strategy for expanding interdisciplinary collaborations and communications at all levels and in all aspects of health care for humans, animals, and the environment [17]. To comprehensively understand the impacts of climate change, One Health concepts can be applied to acknowledge the interdependency between people, animals, plants, and the environment, and develop appropriate interventions for optimal health outcomes [18]. While existing studies have explored the effects of climate change on pastoralist communities in Africa, there is a specific need for research that examines how these impacts are perceived through the One Health lens, particularly in Ethiopian pastoralist regions. This study aims to qualitatively explore how these communities perceive and understand the effects of climate change on the environment, animal health, and human health, using the One Health concept to better understand the interconnections between these factors. The study’s findings serve to enhance comprehension of the potential threats and challenges faced by pastoralist communities, provide a foundation for future research, and inform policies and actions to improve their well-being and livelihoods.

## 2. Methods

### 2.1. Study Setting

At the time of this study, there were 12 regional states and two chartered cities in the Ethiopian government administration. Of these, four regional states (Afar, Oromia, Somali, and Southern Nations, Nationalities and Peoples Region (SNNPR), have pure pastoralist communities that practice little of no agriculture. Together, the Afar, Oromia, and Somali regions contain more than 90% of the pastoralist population in the country. Cattle, camels, goats, and to some extent sheep are the principal livestock species that are reared by Borena pastoralists in the Oromia region, whereas in the Afar and Somali pastoral areas, the camels, followed by goats and sheep, are the most important animals [19].

This study was carried out in two purposively selected districts in each of the three regions (six in total): Elidar and Mille districts in Zone 1 (Awsi Rasu) of the Afar region; Moyale and Yabelo districts in the Borena zone of the Oromia region; and Moyale and Deka districts in the Liben zone of the Somali region. These areas commonly experience conditions of low precipitation and face water scarcity and desertification. The overall biophysical profile of the districts reflects arid and semi-arid climates, posing unique challenges in terms of climate and terrain. The districts were selected based on proximity to the road, with additional security considerations in Afar due to active conflicts in districts bordering the Tigray region during the study period. The selection of the kebeles (the lowest administrative unit in Ethiopia) was based on road access to the villages or at least within walking distance.

### 2.2. Study Approach

A qualitative study was conducted to explore the impacts of climate change on the environment and food insecurity, along with the implications for the health of humans and animals. A descriptive qualitative content approach was used. This approach is an appropriate method for systematically describing the meaning of qualitative data collected through interviews and focus group discussions and present in words and themes, which makes it possible to draw some interpretation of the results [20,21].

### 2.3. Participants and Recruitment

The Focus Group Discussion (FGD) participants were community members, who were life-time residents of the study areas and were recognised by the community as being knowledgeable on the phenomenon of climate change. Only male representatives were included, despite female representatives being initially planned to be included in the quantitative component of the same project. Unfortunately, we were unable to include them and continue the project due to security threats and the ongoing conflict in the regions or along the route, during the data collection period. KII participants were employees of the health and agriculture offices in the selected districts. To recruit KII participants we consulted the heads of the government offices and asked them to nominate people who were knowledgeable on the phenomenon of climate change.

Research assistants, guided by field facilitators, visited the study settings, and purposively recruited participants using the maximum variation method, ensuring a broad representation of perspectives by recruiting participants who differed significantly in terms of age, socio-economic status, and geographic location. They also employed snowball techniques, where the potential participants were asked during the initial contact if they knew of others who could enrich a discussion on the phenomenon of climate change.

### 2.4. Data Collection

A total of 12 FGDs, four in each region, comprising an average of 10 participants per session, were conducted with the community representatives. In addition, 13 KIIs with the district human and animal health and agriculture experts were carried out. We used semi-structured guides with open-ended probing questions to facilitate the discussions. All of the interviews were conducted in the local languages of the respective regions. The interview guides were developed to explore the local understandings of the causes and manifestations of climate change, as well as its impacts and coping mechanisms. The final number of interviews took into consideration the saturation points at which information redundancy was determined. This was achieved after reviewing the field notes and listening to the audio to observe preliminary findings and identify the areas to be further explored.

Three research assistants, experienced in qualitative data collection, and with listening, writing, and speaking skills in the local languages in each region, were recruited and trained. A note-taker, who recorded a summary and the nature of the discussion as well as non-verbal communications including gestures and facial expressions, was present for all the FGDs. All FGDs were conducted in a noise free environment, under the shadow of trees in the local village, whereas the KIIs were conducted in the respective offices of the experts.

### 2.5. Data Management and Analysis

For the analysis, audio recordings were transcribed verbatim and translated into English by an experienced translator. The translated text was then read and re-read by the lead researcher (MT) and a research assistant to code and define the themes and categories guided by the objective of the study. Field notes, which were based on the expansion of the notes taken during interviews and FGDs, were reviewed in order to understand the context. A codebook was prepared after coding two of the transcripts and was then reviewed by the lead author and one co-author (MK) before starting the coding. Thematic analysis was carried out using ATLAS.ti version 8.0 software (Scientific Software Development GmbH, Berlin, Germany) to code the transcripts based on the codebook. We present the findings using the main categories and providing continuous text to draw some interpretation of the results. According to Schreier [20], the qualitative content analysis approach gives the opportunity to choose the aspects on which a researcher wants to focus during the analysis. Following that, the codebook was used as a framework to build our coding frame and select the main categories on which to focus. Inductive approaches were used to create sub-categories, based on the meanings emerging from the data.

### 2.6. Trustworthiness

The trustworthiness of the data was ensured by applying different approaches, following Lincoln’s and Guba’s four criteria, namely credibility, dependability, confirmability, and transferability [22]. Data were collected from different sources using FGDs and KIIs in three different pastoralist regions to assure data triangulation by person and place. In addition, there were debriefing sessions every day during data collection. Efforts were made to ensure consistency between the data before and after the translation via a back translation of two files per each language. We involved three different data collectors and two independent coders to read three selected transcripts and identify the categories, to ensure the dependability of the findings. Data reflecting common views of FGD participants were quoted verbatim, with references to the sources of the excerpts extracted from the data. To ensure transferability, we utilized a substantial description by providing the adequate details on the study site, the participants, their recruitment, and data collection and analysis, in the methods section.

## 3. Findings

### 3.1. Participants’ Information

In this study, 120 community representatives, 40 from each region, participated in 12 FGDs, with ages ranging from 35 to 80 years (average of 58 years). All were married and had an average family size of eight. In terms of education status, 30% did not attend formal education while 48% and 22% attended elementary and high school, respectively. Thirteen key informant interviews were undertaken with experts in human nutrition (6), animal health (5), natural resource management (1), and disaster risk management (1). All except one of the key informants were males.

**Figure 1:**
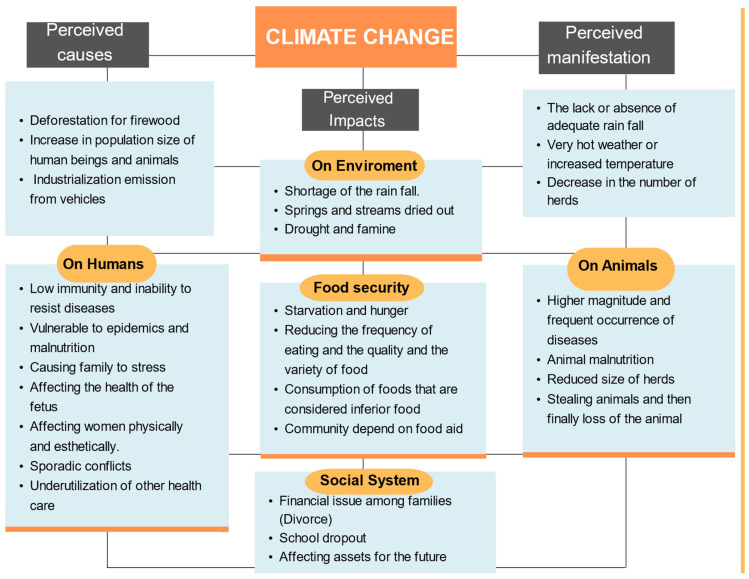
Summary of main and sub-categories.

Five main categories emerged from the analysis, with a further five sub-categories emerging under the third category. Figure 1 shows the summaries of main and sub-categories (Figure 1).

### 3.2. Perceived Causes of Climate Change

Participants of the FGDs perceived that climate change was caused mainly by deforestation. The reasons given for deforestation were the usage of wood for house construction, selling firewood, or making charcoal.

“The main one is deforestation. Previously, there were indigenous trees in this area. But now they are damaged and cleared by human beings. We use wood for construction of houses and cooking mainly but some sell firewood and even prepare charcoal in hiding.”

MD FGD P9.

Others mentioned the increase in human and animal populations as causes of climate change. There were also participants who mentioned the impact of climate change, like shortage of rain and occurrence of floods as the causes. In addition, two participants indicated that they had heard in the media that industrialization, vehicles emissions, and other economic activity like construction were the main contributors to climate change.

“I heard in the media that climate change is because of the industrialization, cars and other constructions…”

HW FGD P2

### 3.3. Perceived Manifestation of Climate Change

For the participants in all three regions, manifestations of climate change are related mainly to the lack or absence of adequate rainfall and very hot weather or increased temperature. They found that the amount of rain and the usual periods of rainy seasons have changed, with very short rainfall, or no rainfall at all, at the time when it usually rains more frequently, in the last three decades. For them, no or little rain in the usual rainy season means drought and famine in the following season. This affects their livelihood, as they rely on animals which require access to grazing land.

“Climate change is [manifesting as] changes to the air, dryness of the water sources like rivers, ponds, local water reservoirs in our kebele and other neighbor kebeles in this woreda”

ED FGD P10

The decrease in the number of herds, especially camels and goats, was also stated as a manifestation of climate change. For the community, very hot temperatures, that their animals could not tolerate, exist because of climate change-induced extreme temperatures.

“The number of livestock per household has been decreasing. I can take my family; we had more than one hundred fifty goats and sheep but currently we have only around sixty or sixty-five goats only. I think this is a good example to see the manifestation of climate change in this woreda or kebeles of the woreda.”

ML FGD P10

One of the participants from Borena zone in Oromia also explained the timing when they started observing the manifestations of climate change by aligning it with their own cultural calendar, as follows:

“It started at the ear of Abba Gada Boru Madha. That means it is about ten years since it started. It was in Ethiopian calendar year 2003 (corresponding to 2011 in the Gregorian calendar). There were trees and grasses ten years around this area, but we do not have them now.”

HR FGD P4

### 3.4. Perceived Impacts of Climate Change

This category describes the analysis of the impacts of climate change on the environment, food security, and health of animals and human beings. In addition, impacts of climate change on the socio-economic and cultural aspects were identified. 

#### 3.4.1. Impacts on the Physical Environment: Feed and Water Sources Disrupted

Climate change has had a severe impact on the environment, including land, air, water, trees, and natural vegetation. A significant issue related to this is the shortage of rainfall, which has resulted in multiple problems for communities. Even during the usual rainy seasons, there has been a lack of rain, especially in the last two decades. Springs and streams dried out before the expected months and failed to replenish, causing concerns over water shortages and the associated dryness, that can lead to drought and famine in the subsequent seasons. One FGD participant highlighted the situation as follows.

“Before 20 or 30 years, we had enough water supplies for our cattle and seasonal rivers and water reservoirs also reserve water for a long duration. But now, the temperature becomes high and there is not enough rainfall around this kebele and the whole woreda too.”

ED FGD P4

**Figure 2:**
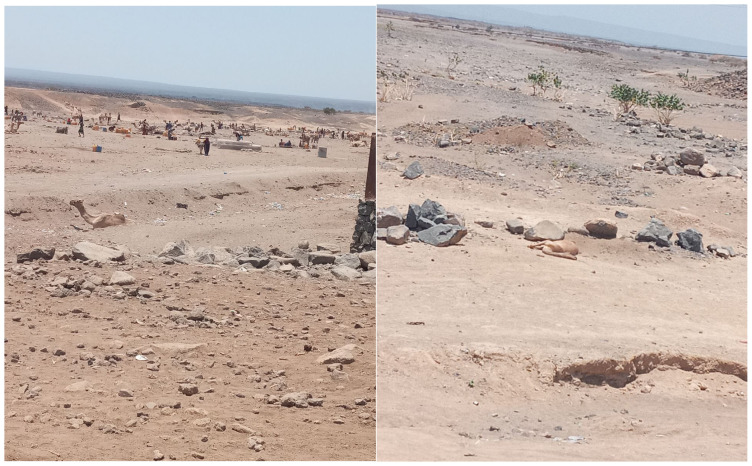
Queue for fetching water and carcass of a dead camel (Photos taken during the field visit in Afar region), 2021, Ethiopia.

During field data collection, our research team witnessed long queues surrounding a deep borehole in Elidar, Afar. The borehole had very little water and there were visible carcasses of dead camels near it. The participants claimed that this was due to the shortage of water and the high temperature in the area. The shortage of rain has not only affected the water availability for humans and animals, but also the growth of desert bushes and grasses in the localities, particularly in Afar. According to the discussants in all three study areas, the environment has become non-conducive for both domestic and wild animals. They have observed the recent extinction of indigenous trees, grasses, and other food sources for their animals. As a result of the shortage of rain during the usual season, the community has been forced to migrate in search of animal feed. This problem has worsened recently, especially in the last three years (Figure 2). 

A 47-year-old person from Afar described it as follows:

“Yes…not only wild animals, but the desert bushes are also decreasing from time to time and some tree species which were abundant in the area before are now difficult to find.”

ML FGD P3

Another older man from Borena, Oromia explained this as follows:

“There is no rainfall this year. This has caused dryness. It only rains for one month two times… There is no growth of trees and plants.”

MD FGD P8

The recent desert locust invasion in the pastoralist regions was another environmental threat perceived to be associated with climate change. The participants mentioned that the locusts affected the already diminishing green plants and small grasses, leaving the environment unsuitable for their animals’ survival.

“Previously when there was rain there would be grass growing. Now due to the presence of desert locust, the desert locust will eat the immediately grown grass and there is nothing left for the animals.”

EG FGD P3

#### 3.4.2. Impacts on Food Security: Shared Vulnerability of Humans and Animals

Food insecurity, characterized by a shortage of quality food, is a critical issue affecting pastoral communities across all three regions. These communities rely heavily on dairy products and the sale of animal products for sustenance. The shortage of rain has dramatically impacted the grazing land and the availability of water for their animals, leading to a decrease in herd size and animal product production. This shortage has been particularly prominent in the past two decades, exacerbating the issue of food insecurity in these communities.

A participant from Afar described it as follows:

“If there is plenty of rainfall in the area there will be enough pasture lands and drinking water for the cattle. So, the result is clear good quality milk, meat and good price for the goats and sheep. The effect is clear for us (pastoral community) because our life is directly affected by climate change for the last ten years here in this kebele as well as in the woreda.”

ED FGD P10

Based on the insight of a natural resource management expert in a district of Somali region, the community’s overall food security is predominantly reliant on the climatic conditions, specifically on the amount of rainfall.

“The relationship between climate and food availability is highly observable. If the climate condition is good, it means there is enough rainfall and good temperature, thus, food security for animals and humans will exist. This will in turn lead to the good production of milk and milk products, good meat supply and fair price for the cattle in the market centers. These all yield for the good livelihood of the pastoral community of the woreda.”

SMY KII NRP

The overall shortage of food and water for the animals, and the resulting shortage of food, lead to starvation and hunger in human populations, and to associated malnutrition among children. All participants indicated their fear of the increase in the magnitude of the problem over time, and especially those in Borena areas were afraid of drought happening in the region.

“…Last time, there was rain for two to three days, and then it disappeared. Therefore, we are now afraid of the impact of climate change on us and on our animals. The sky is not cloudy yet and that means there will not be rain. Cattle started dying and we fear that it will get worse, and drought will happen.”

MD FGD P2

According to the participants, not only the amount of food has decreased, but also the frequency of eating, the quality, and the variety of food has been affected. Some of the community members have started consuming foods which are considered inferior by cultural norms. One participant mentioned that the shortage of food in the area led to the emergence of a new practice of selling camel milk, which had never been practiced before. He stated it as follows:

“I understand the relationship between climate change and food availability because before twenty thirty years ago the community did not sell Camel Milk but nowadays one liter bottle of milk sells fifty or sixty birrs. This is the real example to show the high relationship of climate change and food availability. Climate conditions directly affect the living condition of the community.”

ELD FGD P9

The overall shortage of dairy products, decrease in the herd size, and the resulting food insecurity in some districts of the study areas made the community depend on food aid from the government Safety-Net or on food support from NGOs for the survival of their families.

“The WFP is availing us food. There is also relief aid for some of the community members. Some others get safety net food program… Save the children are helping us with education, water sanitation, MCH (maternal and child health), relief and nutritional interventions.”

SDS KII DRM

However, participants stated that the absence of aid during the COVID-19 pandemic exacerbated the effects of food insecurity due to the lack of support from the government and NGOs. They also mentioned that, during the time of our data collection, there was no active support, for both humans and animals in the community.

“Previously there were NGOs who donate food for animals. They are not helping us now. The government has forgotten them. If the rain began raining the community wouldn’t even seek the support of both the government and the NGOs.”

GF FGD P2

They also added problems related to animal malnutrition, i.e., wasting and stunting, reduced production of milk and its products, animal migration, stealing animals and the subsequent loss of the animal by the owner, and reduced size of herds, particularly cows, sheep, and camels. However, the number of goats has increased. This is because the frequency of reproduction period of goats is shorter than camels and cows.

A key informant from Yabelo Woreda said that “The impacts of climate change on the goats are lack of leaves and water. They become wasted due to shortage of food and water. They are wasted because they are traveling long distances. So are the cattle and camels. The milk and its products will be reduced at the same time.” OYB KII DRM

#### 3.4.3. Impacts on Animal Health: Livelihood Base Affected

According to discussants and animal health experts in the areas, animal health problems perceived to be associated with climate change have impacted animals in multiple ways. Diseases linked to climate change, though not all experts believe they are related, include anthrax, brucellosis, peste des petits ruminants (PPR), foot and mouth disease, lumpy skin disease, contagious caprine pleuropneumonia (CCPP), diarrhea, intestinal parasites, pasteurellosis fulminant respiratory disease (PFR), skin infections, allergies, liver diseases, and diarrhea. Although vaccines exist for some diseases like CCPP and brucellosis, high temperatures and feed shortages have worsened the fatality burden. Animal health experts noted that not all these diseases are attributed to climate change.

“This is the list of diseases for goats, sheep, and cattle. We don’t think that all of them are caused by climate change. The diseases that are caused by climate change are the plague. The others are FMD (foot and mouth disease), LSD (lumpy skin disease), CCPP (contagious caprine pleuropneumonia), and pasteurellosis fulminant respiratory disease (PFR) for cows.”

OMY KII AHE

Coupled with the absence of properly functioning animal health posts and the necessary drugs, the burden of climate change on animal health is perceived as the main challenge for the pastoralist community. Attempts by the local community to construct an animal health post were unsuccessful due to the lack of an animal health expert and the necessary drugs.

“In this Kebele, an animal health post was established in 2004, yet it remains non-functional, posing a significant issue for us as the main problem we face. They should deploy animal health physicians to address this urgent need, as cattle are dying and the post lacks both drugs and human resources.”

MD FGD P7

For them, the loss of camels is an intolerable situation due to its adverse consequences on the economic and socio-cultural aspects of their livelihood.

“Our life is dependent on camels as we get better milk from them, large amounts of meat, we use them for transportation of ourselves or to get water from long distance. One’s richness is even based on the number of camels, as they are very expensive. Now, we can see the dead body of a camel that died before three days due to lack of water.”

ED FGD P1

In addition to domestic animals, the impacts also resulted in the death of wild animals, as indicated below:

“We observed death of wild animals like antelopes in our area.”

ML FGD P5

#### 3.4.4. Impacts on Human Health: Transgenerational Deterioration

Under this category, the participants explained human health problems in association with food insecurity, inaccessibility of drinking water, and animal health, and how these affect resistance to disease. The respondents from the health sector believed that the overall shortage of food for the mothers and their children could lead to low immunity and inability to resist diseases. This made children vulnerable to epidemics of measles and poliomyelitis, as well as illnesses like diarrhea, skin and ear infection, weakness, and anemia. According to the discussants, mothers are affected due to climate change in multiple ways where gendered roles like feeding and domestic chores affected them both physically and emotionally. Lack of drinking water in the nearby area forces the mother to go to other villages and travel long distances, taking hours to fetch water for the family. Lack of adequate food and not being able to feed the children and the rest of the family is stressful for them. One of the participants from the Somali region stated that:

“The impacts of climate change on the health of women are many. They travel long distances to fetch water which may take seven hours to get to the water source. These women have no adequate food. When there is not adequate food, she will be harmed. They lack resistance to the illnesses when they encounter illnesses.”

SDK KII NP

The impact on children starts from the time of conception, due to a poor maternal diet linked with the shortage of food at the family level and an inadequate intake of food affecting the health of the fetus. The participants were aware that when the mother does not get adequate food, the fetus will be thin and unhealthy after the delivery and during the period of exclusive breastfeeding. The problem continues when the child starts complementary feeding, and during and/or after the time of weaning. This is also linked with the shortage of animal milk, and associated with the inability to purchase other food groups because of a low family income caused by the low market price for their animals. A human nutritionist from the Elidar district explained it as follows:

“There is a shortage of food in the community and some community members are displaced to other kebeles and woredas. Malnutrition problems, waterborne disease and other related health problems have occurred many times in our kebeles and causing death of children.”

AED KII NP

Another key informant from Oromia added the following when describing the effects of the scarcity of drinking water, the related waterborne diseases in the communities, and the increased burden of diseases from drinking contaminated water, which the women fetch from unprotected sources:

“The water is not pure. So, human beings including children are exposed to acute watery diarrhea, dysentery, skin allergies and infections, poor growth and development of children, malnutrition, and respiratory infections like common cold, SOB and pneumonia.”

OBY KI NP

He also added that traveling long distances to fetch water affected the women physically. It is tiresome for most, leading to fatigue and stress, and associated with a poor quality of life.

“Since women travel long distances to get drinking water, they become weak and fatigued. Lack of water further causes poor hygiene [cleanliness] of the women. Itching sensation of the skin of pregnant women has also occurred due to climate change.”

OBY KI NP

Indirectly, the impact of climate change among the pastoralist community is linked with the underutilization of other health care services, even including the utilization of maternal and child health services. This is because of the lack of money to cover paid service fees, transportation, and other related costs, including treatment of sick children and mothers. For them, the only source of income is livestock husbandry, and the loss of animals or a low market value means no income.

“We know some services for the mothers and children are free, but we could not get money to cover transportation cost and buying some medicines which are not available at the health centers. Some of us may not be afford for it and not go while our kids are sick and that even lead to death of some children in this area”

AR FGD P3

#### 3.4.5. Impacts on Social Systems: All Rounded Effects

In this category, we presented the impacts of climate change on the social system which includes the existing social interactions and interrelationships between individuals, groups/families, and institutions, based on shared norms and values. We also considered the economic system and the cultural aspects as part of the social systems.

In normal circumstances, the husband is responsible for generating income, usually by selling animals, and providing money for the livelihood of the family. However, this role becomes challenging due to not being able to sell the animals, due to undernutrition or disease. In some situations, disputes following this financial issue ended up with divorce and broken families when the husband could not avail himself with the necessary food and other basic needs for their family. The participants mentioned that aid from the government or other donors was being used in such times, but was not available at the time of this study, worsening the situation.

Climate change is perceived to have increased school dropout rates, due to lack of food and the related fatigue of traveling long distances to school; sickness of the student; and/or the additional responsibility of traveling longer distances to graze the animals and to fetch water.

“Due to shortage of food the students are not going to school. They are not attending their school. … They are even fatigued. They are unable to walk to school.”

EG FGD P8

Participants elucidated the economic repercussions from the various perspectives intertwining with the social fabric. The inability of animals to generate revenue in the market intensified the economic strain. For some community members, the heightened market value of essential daily needs became intolerable, impacting their overall well-being within the social system. The elevated market prices also contributed to households reducing the frequency of meals and struggling to afford necessities, including clothing for themselves and their children.

“Our herds are dying and those living are also becoming weak. We cannot sell them in the current market, and we could not get adequate milk and other milk products. On the other hands the market prices of everything we buy is highly increasing. Life is becoming very difficult to live here in the recent years”

DR FGD P7

Conversely, participants highlighted that the reductions in their herd size and the deterioration of their animals had repercussions on their family wealth and future assets, due to both low market prices and animal mortality. Instances of deceased camels and cattle were evident during the field data collection, particularly in the Afar and Borena zones of Oromia. FGD participants expressed concerns that the prospect of family income and future livelihood is jeopardized.

“The price of camels, cattle and other animals dropped at the market due to their starved condition caused by food scarcity. This poses significant challenges for us (pastoral communities) striving to generate sufficient income to procure essential goods from the market.”

ED FGD P9

Culturally, it was common to share food and other resources within the community members. However, participants indicated that the lack of adequate food for their own households, due to climate change, affected the existing culture. Now, they complain that it is impossible to share even with relatives and close families, thus, the poor started begging others for their daily survival, while others migrated to the urban settings, searching for new jobs or to beg, leading to pastoralist dropout. There is no other means to survive when they lose their assets (the animals), since no one wants to lend money. A FGD discussant reflected on this as follows:

“Others may lend you when you have cattle. In addition, those cattle must be healthy and fat. When there is climate change the cattle become thin and starved. During this time, those who have money may not lend you.”

FL FGD P1

According to discussants from Borena, sporadic conflicts occur in the competition for the limited water and grazing land resources and/or, rarely, cattle raiding by other pastoralists from different tribal or neighboring countries can also occur. This is also linked with the risks of land degradation in the localities, as they mention that resource competition among the different groups is linked with the grazing land.

“Conflict in grazing land use and stealing animals is common during the summer season. There is no theft in the winter season. We have encountered problems while traveling longer distances like 5 to 6 KM. We may lose our cattle when we are herding them. They [raiders] may even kill the herder and that may lead to tribal conflict and more casualties.”

MD FGD P9

### 3.5. Coping Mechanisms: Searching for New Livelihood and Occupation

This category describes the measures local communities take to overcome the impact of climate change. Pastoral communities employ various strategies to cope with these impacts, ranging from storing grass for animals to engaging in new economic activities such as selling wood and charcoal and becoming daily laborers. Additionally, they undertake environmental recovery attempts like afforestation and resource conservation, as well as migration and seeking aid from the government or NGOs.

In places like the Borena zone of Oromia, pastoralists used to store grass during the rainy season to ensure adequate nutrition for their animals. This was especially common after experiencing food shortages during the dry seasons, which they believe are attributed to the impacts of climate change. In other areas, lacking sufficient natural grass, they gather maize stems to mitigate the effects. However, such practices are limited to certain geographic areas, and the stored grass may not be suitable for all animals, such as camels, which do not consume it. Unfortunately, they did not have adequate stores during the time of data collection for this study. A participant in a FGD expressed this sentiment:

“We collect and utilize grass, and we use maize branches to feed the cattle. When climate change exacerbates, we sell the cattle and endure the season. We purchase maize bread to feed our cattle. Currently, the duration of rainfall is minimal, and there is a lack of grass due to poor growth.”

MK FGD P1

Participants stated that the community began engaging in selling firewood and/or preparing charcoal by cutting the already diminishing trees to gain income and ensure the survival of their family. According to the respondents from the Borena zone of Oromia, such types of activities were uncommon, except for the rare practice of some members of the community living along the main road. In the past, the community did not make charcoal even from a tree falling by itself; their herd was enough to have food for the family and to cover other costs.

“There are also people that collect and sell firewood. They also make and sell charcoal which was not common in the past time”

AR FGD P1

In addition, others migrate and settle in the nearby cities or the bordering countries searching for jobs. Some leave their village and migrate to other places, where they might have close family or other relatives to help provide food and other life-supporting commodities. However, this is not an option for children, women, elders, and those with disabilities, as traveling long distances in very hot weather is difficult for them. Even the family members who take care of such vulnerable groups might not migrate, even though they might want to.

“Some of our neighbors went far to other kebeles and there are others go to nearby towns to search for any job. Even we heard there were others begging in Addis Ababa and sending money for their families.”

HR FGD P5

In some places/villages, community level attempts have been made to protect the environment, by implementing local reforestation of the degraded areas and trying to use the existing resources in an efficient way.

“We are working to preserve the lands, mountains and water source areas through safety net program.”

ED FGD P1

### 3.6. Suggestions for Adaptation

In this category, we presented what the participants of the study suggested to be effective when moving forward. In addition to supporting their local environmental recovery efforts, by implementing reforestation to mitigate the impacts of deforestation, they suggested other solutions, with an emphasis on addressing the shortage of food for themselves and their animals.

Regarding the problems which take priority, all the participants have proclaimed the lacking food availability for humans, as well as the animals, as the main issue. They believe that their survival is the first and foremost issue to help the animals and to protect the environment. They justified that traveling long distances to feed their animals and physical work to protect their environment needs their survival. They requested availability and continuity of food aid by the NGO’s to assist their daily survival. They indicated that such aid was available before, and was interrupted recently. To this end, they also recommend strengthening the safety net program (government program aiming at reducing food insecurity vulnerability by providing economic opportunities and building resilience to crises, through cash transfers, public works, and nutritional feeding programs) at the community level.

“We cannot live without food and water. We have a problem of food for ourselves and for our animals. The government or other NGOs should avail water and food for us…. The safety net programs should be increased.”

HW FGD P3

For them, the lack of an early warning and alerting at the community level by the local government system needs improvement. Experts suggested the need for an early warning to better prepare an mobilize the local community, and that helping them be prepared for an emergency is important. Their life is full of fear, and they are worried about the occurrence of hunger in the following season, as they know there was inadequate rain during the time it usually rains.

“There should be early warning and community engagement in the emergency response preparedness. The problems are mostly addressed after loss of animals or even after death of children.”

AED KII HN

They also suggested that the government and other aid organizations need to increase the reliefs and the nutritional interventions available for children. They repeatedly raised that the children are the most vulnerable during droughts; therefore, they suggested that provision for them should take priority. In a normal situation, the community gives priority to feeding the children; however, a shortage or lack of food could make them unable to feed them properly.

“Now, we have no food, and we request non-governmental organizations to give us food specially nutrients for children and pregnant women.”

DS FGD P6

Availing health care provisions, and strengthening the existing ones, for the participants and their animals, was emphasized during the discussions. They complained that the services provided during the time of such drought for pastoralists needs to be improved, compared with other areas. They also indicated that the areas are hard to reach even in normal conditions, and that the current issues exacerbated the problem.

“We need the government and others to provide us with good health care and medicine for ourselves and our animals.”

ED FGD P7

## 4. Discussion

This study aimed primarily to explore how the pastoralist community viewed the impact of climate change on the physical environment, food security, and health of animals and humans. The findings shed light on the perspectives of the participants regarding the perceived causes of climate change; they connect it with the widespread clearing of indigenous trees for various purposes, including house construction, firewood sales, and charcoal production. Some participants also acknowledged global contributors to climate change, such as industrialization and vehicle emissions.

The findings underscore the multifaceted impacts of climate change on various dimensions of the participants’ lives. The severe consequences on the physical environment, encompassing land, water, trees, and vegetation, are vividly articulated. The scarcity of rainfall, which the participants believed was due to climate change, across all regions, even during the expected seasons, disrupts the water sources and creates water shortages for humans and animals. Witnessing visible carcasses of dead camels around a borehole in Elidar, Afar, exemplifies the direct link between water scarcity, high temperatures, and adverse impacts on both domestic and wild animals. The natural environment of the main pastoralist regions of Ethiopia are located in the peripheral lowland settings, with arid or semi-arid temperatures, rain shortages, and recurrent droughts, now aggravated by the impacts of climate change [23]. As voiced by the participants, the shortage of drinking water and water for their animals, which resulted from the lack of, or insufficient rainfall, coupled with an increase in extreme heat, is the main problem that affected their livelihood. Participants of the three regions emphasized the recurrence of failed rain seasons and the related droughts in recent decades, compared with the past, and all, especially in Borena, were in fear of drought during the time of data collection.

UNICEF’s press release, a few months after our data collection, highlighted the drought’s devastating effects, especially among pastoralists in Oromia and Somali regions. Over 4.4 million people faced critical water shortages, with 225,000 malnourished children and 100,000 pregnant and breastfeeding women needing urgent nutrition support [24]. Not capitalizing on the participants’ pre-existing fear of drought was a missed opportunity for community alerting, disaster risk reduction, and emergency preparedness. Utilizing indigenous knowledge for effective mitigation strategies, including establishing a forecasting system, needs to engage the local communities [25].

The implications of climate change extend to food security, revealing a shared vulnerability among pastoralists and their animals. Decreased rainfall significantly impacts grazing land, leading to reduced herd sizes and animal product production, exacerbating food insecurity in the regions heavily reliant on dairy and animal products. Participant narratives underscored the interconnectedness of climate conditions, food availability, and overall pastoral community well-being, potentially leading to undernutrition [25,26] associated with the increased susceptibility to infection [27] or vulnerability to chronic diseases and mortality [28]. In line with these findings, a study among Hammer pastoralists in Southern Ethiopia emphasized malnutrition because of climate change, especially affecting vulnerable groups like children, due to a decline in animal milk availability [16].

The findings of this study showed the impacts of climate change and the resulting food insecurity on social systems and encompassed the economic hardships, cultural shifts, and conflicts over the diminishing resources within, and bordering the countries. The traditional role of husbands as providers becomes challenging due to the inability to sell animals, leading to financial disputes and broken families. In some localities, school dropout rates rise, due to factors such as lack of food, fatigue from long travel distances, and increased responsibilities related to herding animals and fetching water. Economic aspects suffer, as inflation, decreased meals, and inability to buy essential items coincide with diminishing animal resources, contributing to a precarious future for these communities. The emergence of the COVID-19 pandemic, and the subsequent national lockdown to prevent disease transmission, interrupted the aid from the government and from NGOs and significantly exacerbated these impacts. The shift to the consumption of foods considered to be inferior by the community could also affect the social status and self-view which might lead to lack of social acceptance and further mental health problems [29].

In some areas, the lack of food and loss of assets (the animals) is forcing the pastoralists to migrate to urban settings searching for a job, or to live on streets by begging. This is the unintended impact of climate change on urbanization and urban environment. This may have consequences on the already existing unemployment in the urban labor market and on the living costs, including housing, in the main urban settings [30]. It could also affect the long-term career path of the youth and the future development of the country.

The engagement of local communities in selling firewood and charcoal to generate income in some areas could further exacerbate the problem. The cutting of more trees for the those means of income and for survival will leave the environment, which is already vulnerable, more susceptible to climate change impacts by triggering flash flooding [31]. The eroded soil will not retain water and support the growth of plants, including the grasses for the animals, and will result in aggravating the impacts of climate change and making them susceptible to drought conditions [32]. The recent drought in the Borena zone of the Oromia and Somali regions [24], soon after this study was conducted, indicates the contribution of already existing impacts.

This is a qualitative study, conducted among pastoralist communities in three regional states, and cannot be generalized to the national level. However, the regions hold over 90% of the country’s pastoralist communities. The findings will aid the understanding of the impacts of climate change and to planning of multidisciplinary interventions in the future. Another limitation is related to the regions’ location in arid and semi-arid low-land areas, and their historical experience with drought. Distinguishing between natural climate variability and the influence of long-term climate change poses a challenge, making it difficult to fully attribute the impacts solely to climate change.

## 5. Conclusions

In conclusion, this study highlighted the impacts of climate change on pastoralist communities, revealing interconnected challenges in the physical environment, food security, and animal and human health. Participants identified local practices of deforestation(e.g., for house construction and fuel production) and acknowledged global factors, such as industrialization and vehicle emissions, as contributors to climate change. Food insecurity, driven by the diminished grazing land and animal resources, poses threats to nutrition and health, particularly among vulnerable populations like children, and potential long-term consequences on the health and well-being of future generations. Economic hardships, cultural shifts, and conflicts over diminishing resources were identified as social impacts, with migration to urban settings emerging as an unintended consequence, affecting both the urban labor market and the future trajectory of the country. Early warning and community resilience programs should be designed to increase the community’s capacity to resist the effects of the problems, and to reduce the burden of the consequences. We recommend the local design of comprehensive, community-driven strategies to address the multifaceted challenges posed by climate change in pastoralist regions, aiming to avoid the exacerbating environmental vulnerabilities already susceptible to climate change impacts. Further quantitative research needs to be conducted to estimate the impact of climate change.

## Data Availability

The original raw data used in this study is available from the corresponding author and can be presented upon reasonable request.
